# Assessment of Federal Value-Based Incentive Programs and In-Hospital *Clostridioides difficile* Infection Rates

**DOI:** 10.1001/jamanetworkopen.2021.32114

**Published:** 2021-10-29

**Authors:** Mohammad Alrawashdeh, Chanu Rhee, Heather Hsu, Rui Wang, Kelly Horan, Grace M. Lee

**Affiliations:** 1 Department of Population Medicine, Harvard Medical School and Harvard Pilgrim Health Care Institute, Boston, Massachusetts; 2 Jordan University of Science and Technology, Irbid, Jordan; 3 Department of Medicine, Brigham and Women’s Hospital, Boston, Massachusetts; 4 Department of Pediatrics, Boston University School of Medicine, Boston, Massachusetts; 5 Department of Biostatistics, Harvard T.H. Chan School of Public Health, Boston, Massachusetts; 6 Department of Pediatrics, Stanford University School of Medicine, Stanford, California

## Abstract

**Importance:**

Health care facility–onset *Clostridioides difficile* infection (HO-CDI) rates reported to the US Centers for Disease Control and Prevention’s National Healthcare Safety Network (NHSN) became a target quality metric for 2 Centers for Medicare & Medicaid Services (CMS) value-based incentive programs (VBIPs) in October 2016. The association of VBIPs with HO-CDI rates is unknown.

**Objective:**

To examine the association between VBIP implementation and HO-CDI rates.

**Design, Setting, and Participants:**

This interrupted time series study evaluated HO-CDI rates among adults hospitalized from January 2013 to March 2019 at 265 acute-care hospitals.

**Interventions:**

Implementation of VBIPs in October 2016.

**Main Outcomes and Measures:**

Quarterly rates of HO-CDI per 10 000 patient-days, as reported to NHSN by participating hospitals, were evaluated. Generalized estimating equations were used to fit negative binomial regression models to estimate immediate program effect size (ie, level change) and changes in the slope of HO-CDI rates, controlling for each hospital’s predominant method of CDI testing (ie, nucleic acid amplification test [NAAT], enzyme immunoassay [EIA] for toxin, or other testing methods).

**Results:**

The study cohort included 24 332 938 admissions, 109 371 136 patient-days, and 74 681 HO-CDI events at 265 hospitals (145 [55%] with 100-399 beds; 205 [77%] not-for-profit hospitals; 185 [70%] teaching hospitals; 229 [86%] in metropolitan areas). Compared with EIA, rates of HO-CDI were higher when detected by NAAT (adjusted incidence rate ratio [aIRR], 1.55; 95% CI, 1.40-1.70; *P* < .001) and other testing methods (aIRR, 1.47; 95% CI, 1.26-1.71; *P* < .001). There were no significant changes in testing methods used by hospitals immediately after VBIP implementation. Controlling for CDI testing method, VBIP implementation was associated with a 6% level decline in HO-CDI rates in the immediate postpolicy quarter (aIRR, 0.94; 95% CI, 0.89-0.99; *P* = .01) and a 4% decline in slope per quarter (aIRR, 0.96; 95% CI, 0.95-0.97; *P* < .001). Results were similar in a sensitivity analysis using a 1-year roll-in period accounting for the period after the announcement of the HO-CDI VBIP policy and prior to its implementation.

**Conclusions and Relevance:**

In this study, VBIP implementation was associated with improvements in HO-CDI rates, independent of CDI testing method. Given that CMS payment policies have not previously been associated with improvements in other targeted health care–associated infection rates, future research should focus on elucidating the specific processes that contributed to improvement in HO-CDI rates to inform the design of future VBIP interventions.

## Key Points

**Question:**

Was the incorporation of health care facility–onset *Clostridioides difficile* (HO-CDI) rates into value-based incentive programs in October 2016 associated with changes in HO-CDI rates in US hospitals?

**Findings:**

In this interrupted time series study of more than 24 million admissions at 265 hospitals between January 2013 and March 2019, the incorporation of HO-CDI into value-based incentive programs was associated with a 6% decline in HO-CDI rates in the immediate postpolicy quarter and a 4% decline in slope per quarter.

**Meaning:**

In this study, value-based incentive programs were associated with a decline in national HO-CDI rates, which may reflect improvements in infection control practices and better antibiotic and diagnostic stewardship.

## Teaser

This nonrandomized clinical trial examines the association between value-based incentive program implementation and health care facility−associated *Clostridioides difficile* infection rates.

## Introduction

*Clostridioides difficile* (formerly *Clostridium difficile*) is a gram-positive, spore-forming bacillus that can cause serious infectious diarrhea. In 2017, *C. difficile* infections (CDI) were responsible for 223 900 cases in hospitalized patients, 12 800 associated deaths, and approximately $1 billion in attributable health care costs.^1^ Given its substantial health and economic burden, health care facility–onset CDI (HO-CDI) was among the health care–associated infections (HAIs) targeted by US Department of Health and Human Services 2013 Action Plan for prevention efforts.^2^

To help achieve the HAI Action Plan’s 2020 target to reduce HO-CDI by 30%,^3^ the Centers for Medicare & Medicaid Services (CMS) established 2 value-based incentive programs (VBIPs), the Hospital Value-Based Purchasing (HVBP) and the Hospital-Acquired Conditions Reduction Program (HACRP), that link financial incentives or penalties to hospitals’ performance on selected quality metrics, including HO-CDI rates.^4^ HVBP rewards or penalizes hospitals by as much as 2% of total Medicare inpatient payments based on hospital performance on selected quality measures, while the HACRP withholds as much as an additional 1% of Medicare payments from the lowest-performing hospitals.^4^ In October 2016, these VBIPs began including HO-CDI rates reported to the US Centers for Disease Control and Prevention’s (CDC) National Healthcare Safety Network (NHSN) as a component of targeted quality measures.^5^

To date, national HO-CDI rates have not been evaluated in light of VBIP implementation. In this study, we used prospectively collected NHSN data from a large cohort of US hospitals to assess changes in HO-CDI rates associated with VBIP implementation.

## Methods

### Study Design, Population, and Data Sources

We used an interrupted time-series design to examine the association between VBIP implementation in October 2016 and changes in trends for NHSN HO-CDI rates. The NHSN is a comprehensive national surveillance system used by more than 4000 acute-care facilities in all 50 states, Washington, DC, and Puerto Rico. Hospitals prospectively track HAIs using standardized surveillance case definitions based on laboratory and clinical data and report cases to NHSN. Hospitals started reporting CDI laboratory-identified events to the NHSN as part of CMS Hospital Inpatient Quality Reporting Program on January 1, 2013.^6^ We obtained prospective NHSN surveillance data from a cohort of nonfederal acute-care hospitals subject to the Inpatient Prospective Payment System and enrolled in the Preventing Avoidable Infectious Complications by Adjusting Payment (PAICAP) study.^7^ We included CDI surveillance data from all PAICAP study hospitals conducting NHSN CDI reporting from January 1, 2013, through March 31, 2019. We analyzed data from hospitals that reported facility-wide CDI events and excluded hospitals that contributed data only from specific departments.

We obtained hospital characteristics including hospital size, teaching status, type of ownership (ie, public, for-profit, or not-for-profit), location (metropolitan [urbanized area with population ≥50 000], micropolitan [10 000-49 999 population], or rural [<10 000 population]), and percentage of patient-days insured by Medicare or Medicaid from the 2015 American Hospital Association annual survey.^8^ The institutional review board of Harvard Pilgrim Health Care Institute approved this study with a waiver of informed consent because data were deindentified. The study followed the Transparent Reporting of Evaluations with Nonrandomized Designs (TREND) reporting guideline.

### CDI Case Definition

Per NHSN criteria, we defined HO-CDI as laboratory-identified events for stool specimens collected more than 3 calendar days after admission from any inpatient location (regardless of patient signs or symptoms). We defined community-onset CDI (CO-CDI) as laboratory-identified events for stool specimens collected from inpatient locations up to 3 days after admission or from outpatient settings for patients who did not have an inpatient discharge in the last 28 days. We calculated quarterly CDI incidence rates (cases per 10 000 patient-days) for each health care facility by dividing the overall number of CDI events by the number of total inpatient days for the facility and multiplying by 10 000. We excluded any recurrent laboratory-identified CDI and community-onset health care facility–associated events, which are laboratory-identified events collected from an inpatient or outpatient location for patients who were discharged within the prior 28 days.^9^

Due to variations in the sensitivity of different CDI testing methods, hospitals are required to report their predominant CDI testing method on a quarterly basis, defined as the method used for more than 50% of specimens.^9,10,11^ CDC classifies CDI testing methods into three groups: (1) nucleic acid amplification testing (NAAT), including NAAT alone or any testing algorithm that includes NAAT as the final step; (2) enzyme immunoassay (EIA), including EIA alone or any algorithm with EIA in the final step; and (3) other, including cell cytotoxicity assay, toxigenic culture, or another method. For multistep testing algorithms on the same unformed stool specimen, as per CDC criteria, the final test performed was used to determine whether the case definition for laboratory-identified CDI was met.

### Statistical Analysis

We performed descriptive analyses to summarize continuous variables using medians and IQRs and categorical data using proportions and frequencies. We reported CDI rates using weighted mean and SD to adjust for the variation in reported patient-days between hospitals. We used generalized estimating equations (GEE) with robust sandwich variance estimators to fit negative binomial regression models to identify changes in the level and trend of HO-CDI rates associated with VBIP implementation and account for clustering at the hospital level. Terms in the models included time (in quarters), an indicator for program implementation in quarter 4 of 2016 (to assess for level change in CDI rates), the interaction between time and program implementation (to assess for change in slope after program implementation), and the predominant CDI testing method (to account for variable sensitivity of CDI testing methods). Number of patient-days was used as the model offset.

In addition, we explored a 3-way interaction between time, program implementation, and predominant CDI testing method to assess whether CDI testing method modified the program implementation effect. We excluded this 3-way interaction term from the primary model because it was not significant.

We performed 2 sensitivity analyses. First, we evaluated a model that considered the year prior to program implementation to be a roll-in period, as the official announcement of inclusion of HO-CDI as a VBIP target occurred with the publication of the 2016 CMS Final Rule (CMS-1632-F) in July and October 2015, approximately 1 year before program implementation.^12^ Second, we compared trends in HO-CDI to the non-VBIP targeted CO-CDI to understand the degree to which HO-CDI trends might have been associated with actions that are specific to hospitalized patients (eg, better infection control practices) as opposed to actions that would affect both CO-CDI and HO-CDI rates (eg, better diagnostic stewardship). We conducted an interrupted time series model to test the change in the ratio of absolute numbers of HO-CDI to CO-CDI cases over time, controlling for CDI testing method and VBIP implementation and its interaction with time. We considered 2-sided *P* < .05 to be significant, and all analyses were conducted using R version 4.02 (R Project for Statistical Computing) and SAS Studio version 3.7 (SAS Institute).

## Results

### Study Population

Of the 394 PAICAP study hospitals reporting to NHSN between 2013 and 2019 and subject to the Inpatient Prospective Payment System, 129 hospitals were excluded from this analysis because they contributed data only from selected departments and did not report facility-wide CDI events. The excluded hospitals were comparable with included hospitals with regard to region, bed size, teaching status, number of full-time equivalent nurses per 100 patient-days, and the percentage of patient-days covered by Medicare and Medicaid.

A total of 265 hospitals from 47 states and the District of Columbia participated in the VBIPs and consistently reported facility-wide CDI events to NHSN during the study period and thus were included in this analysis. In total, these hospitals reported 24 332 938 admissions, 109 371 136 patient-days, and 74 681 HO-CDI events. Most hospitals were medium-sized (ie, 100-399 beds; 145 [55%]), not-for-profit (205 [77%]), teaching hospitals (185 [70%]), and located in metropolitan areas (229 [86%]) (Table 1). There were no missing data for the variables of interest.

**Table 1.** Characteristics of Study Hospitals Reporting Facility-Wide *Clostridioides difficile* Infection Measures to the National Healthcare Safety Network

Hospital characteristic^a^	PAICAP hospitals, No. (%) (N = 265)
Region	
Midwest	60 (22.6)
Northeast	93 (35.1)
South	68 (25.7)
West	44 (16.6)
Location^b^	
Metropolitan	229 (86.4)
Micropolitan	32 (12.1)
Rural	4 (1.5)
Bed size	
<100	41 (15.5)
100-399	145 (54.7)
≥400	79 (29.8)
Type of ownership	
For-profit	34 (12.8)
Not-for-profit	205 (77.4)
Public	26 (9.8)
Teaching status^c^	
Graduate	121 (45.7)
Major	52 (19.6)
Minor	12 (4.5)
Nonteaching	80 (30.2)
Full-time equivalent nurses, median (IQR), No. per 100 patient-days	0.89 (0.72-1.10)
Inpatient-days covered, median (IQR), %	
By Medicare	49.1 (43.9-57.5)
By Medicaid	21.1 (14.8-25.0)

Abbreviation: PAICAP, Preventing Avoidable Infectious Complications by Adjusting Payment.

^a^ Data on hospital characteristics come from the 2015 American Hospital Association annual survey.

^b^ Metropolitan areas are urbanized areas with populations of at least 50 000; micropolitan areas, 10 000 to 49 999 population; rural areas, less than 10 000 population.

^c^ All hospitals were placed into 1 of 4 categories based on their response to the American Hospital Association annual survey: major teaching hospitals (those that are members of the Council of Teaching Hospitals), graduate teaching hospitals (non–Council of Teaching Hospitals members with a residency training program approved by the Accreditation Council for Graduate Medical Education), minor teaching hospitals (non–Council of Teaching Hospitals members with a medical school affiliation reported to the American Medical Association), and nonteaching hospitals (all other institutions).

### HO-CDI Rates by Testing Method

**Figure 1.**
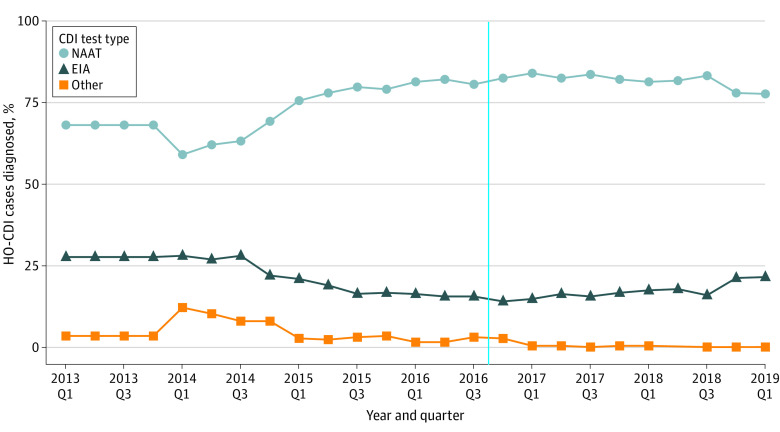
Percentage of Cases of Health Care Facility–Onset *Clostridioides difficile* Infection (HO-CDI) Diagnosed by Different Testing Methods at 265 US Hospitals, 2013 to 2019 Vertical dashed line represents the implementation of value-based incentive payment program in October 2016. No cases were reported for 2018 quarter (Q) 2 in the other testing method. EIA indicates enzyme immunoassay; NAAT, nucleic acid amplification test.

The percentage of hospitals reporting NAAT as their predominant CDI testing method gradually increased from quarter 1 of 2013 (181 hospitals [68%]) to quarter 1 of 2017 (223 [84%]) and subsequently decreased to 206 hospitals (78%) in early 2019 (Figure 1). In general, however, there were no major shifts in the testing methods used immediately after VBIP implementation.

HO-CDI incidence was highest among hospitals that predominantly used testing methods from the other (mean [SD] cases per 10 000 patient-days, 7.42 [3.21]) and NAAT (mean [SD] cases per 10 000 patient-days, 7.22 [3.36]) categories, followed by the EIA category (mean [SD] cases per 10 000 patient-days, 4.95 [2.92]). The GEE model confirmed that predominant use of NAAT and other CDI testing methods was associated with higher HO-CDI incidence rates compared with the EIA method (NAAT: adjusted incidence rate ratio [aIRR], 1.55; 95% CI, 1.40-1.70; *P* < .001; other CDI testing methods: aIRR, 1.47; 95% CI, 1.26-1.71; *P* < .001).

### Association Between VBIP Implementation and the HO-CDI Rate

**Figure 2.**
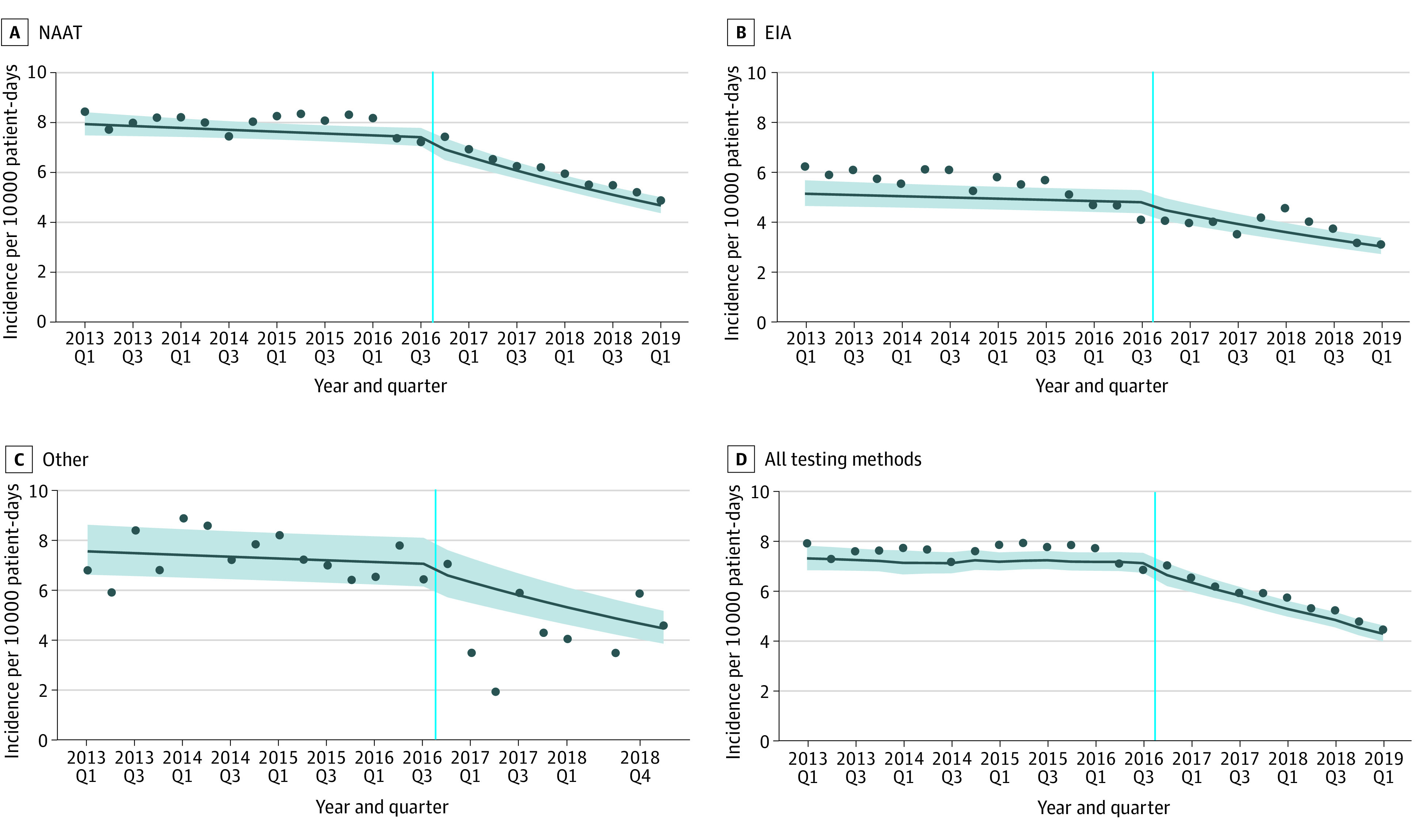
Association of Value-Based Incentive Program Implementation With Observed and Predicted Health Care Facility–Onset *Clostridioides difficile* Infection Rates by Testing Method and Overall at 265 US Hospitals, 2013 to 2019 Circles depict observed health care facility–onset *C. difficile* infection rates. Solid lines represent generalized-estimating equation model-estimated outcomes, with shaded areas indicating 95% CIs. Vertical line represents the implementation of the value-based incentive programs in October 2016. No cases were reported for 2018 quarter (Q) 2 in the other testing method. EIA indicates enzyme immunoassay; NAAT, nucleic acid amplification test.

Regardless of predominant testing method type, there were similar trends of decreasing HO-CDI incidence after VBIP implementation (Figure 2A-C). The overall mean (SD) HO-CDI incidence across all hospitals during the study period was 6.8 (3.4) CDI events per 10 000 patient-days (Figure 2D).

Table 2 shows the results from the GEE model for the association between VBIP implementation and reported quarterly HO-CDI rates. After controlling for CDI testing method, there was a 6% level decline in the HO-CDI incidence rate (aIRR, 0.94; 95% CI, 0.89-0.99; *P* = .01) followed by a gradual slope decline after that (aIRR, 0.96; 95% CI, 0.95-0.97; *P* < .001). Results were similar in the model using a roll-in period for the year prior to VBIP implementation (Table 2).

**Table 2.** Results of the Multivariable Generalized Estimating Equations Model for the Association of VBIP Implementation With Reported Quarterly Rates of Health Care Facility–Onset CDI

Variable	Roll-in period included			
	No		Yes	
	Adjusted IRR (95% CI)	*P* value	Adjusted IRR (95% CI)	*P* value
Time, ie, rate slope before policy implementation	0.995 (0.990-0.999)	.04	1.00 (0.99-1.01)	.94
Policy, ie, change at time of VBIP implementation	0.94 (0.89-0.99)	.01	0.89 (0.82-0.96)	.003
Time × policy, ie, change in the slope after VBIP implementation^a^	0.96 (0.95-0.97)	<.001	0.96 (0.95-0.97)	<.001
CDI testing method				
NAAT	1.55 (1.40-1.70)	<.001	1.52 (1.38-1.67)	<.001
Other	1.47 (1.26-1.71)	<.001	1.44 (1.25-1.66)	<.001
EIA	1 [Reference]	NA	1 [Reference]	NA

Abbreviations: CDI, *Clostridioides difficile* infection; EIA, enzyme immunoassay; IRR, incidence rate ratio; NA, not applicable; NAAT, nucleic acid amplification testing; VBIP, value-based incentive program.

^a^ A model with 3-way interaction for time, policy (VBIP implementation), and CDI testing method was explored, and the interaction term was not significant.

### HO-CDI vs CO-CDI Rates

**Figure 3.**
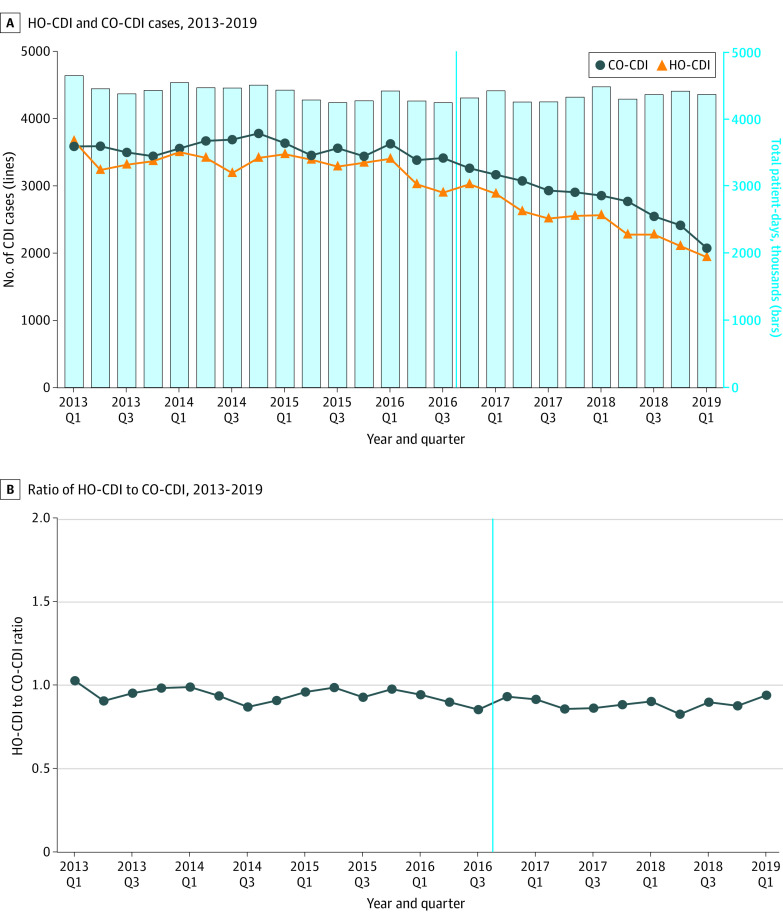
The Aggregate Number and Ratio of Health Care Facility–Onset (HO) to Community-Onset (CO) *Clostridioides difficile* Infection (CDI) Incidence at 265 US Hospitals, 2013 to 2019 Vertical line represents the implementation of value-based incentive programs in October 2016. Q indicates quarter.

The aggregate numbers of HO-CDI and CO-CDI cases among admitted patients showed a large decrease between quarter 1 of 2013 and quarter 1 of 2019 (HO-CDI, 3668 vs 1939; CO-CDI, 3587 vs 2073). Both types of CDI had commensurate trends, with a sharp decline after VBIP implementation, while the number of surveilled patient-days (denominator for HO-CDI rates) remained constant during the study period (Figure 3A). The ratio of absolute numbers of inpatient HO-CDI to CO-CDI cases was constant over time, indicating no fluctuation in the balance between the rates of the 2 types of CDI, even after the implementation of VBIPs (Figure 3B). This finding was confirmed by a regression model that showed no significant association between time, VBIP implementation, and the interaction of time and VBIP implementation on the ratio between the aggregated health care facility to community CDI incidence over time.

## Discussion

The VBIPs are one of the broadest initiatives to incentivize prevention of HO-CDI in US hospitals. In this large cohort study of 265 US hospitals, we found that the implementation of VBIPs was associated with a consistent decline in the rates of HO-CDI infection, regardless of the predominant CDI testing method used by the hospital. Our findings were similar when including a 1-year roll-in period to account for the lag between the announcement of the inclusion of HO-CDI as a VBIP target and program implementation.

The rapid decline in HO-CDI rates we observed after the implementation of VBIPs is consistent with data from CDC’s Emerging Infections Program that showed a decline in both the adjusted and actual HO-CDI rates at 10 US sites from 2011 through 2017, with the greatest reduction starting in 2016.^13^ In addition, standardized infection ratios for CDI among national acute-care hospitals declined between 2016 and 2018.^14^ Turner et al^15^ found a modest decline in HO-CDI rates in a sample of 43 community hospitals between 2013 and 2018 but only after controlling for testing method; the increasing use of NAAT as a testing method in that study might have attenuated the temporal reduction in HO-CDI rates.

To our knowledge, this is the first HAI for which a CMS policy has been associated with improvement. Previously implemented CMS policies and the current VBIPs showed no evidence in reducing the rates of central catheter–associated bloodstream infections, surgical site infections after colon surgical procedures and abdominal hysterectomy procedures,^16^ catheter-associated urinary tract infections,^17,18^ and mediastinitis following coronary artery bypass graft surgery.^19,20^

There are several possible explanations for the apparent association between VBIPs and the decline in HO-CDI rates. First, this could reflect a commitment by health care organizations in reducing the infection rates by taking increasingly aggressive measures to prevent CDI. Adherence to infection control measures, such as handwashing, contact precautions, and patient isolation, environmental screening and cleaning, and the use of sporicidal germicides,^21,22,23^ may have improved over time and are potentially modifiable with hospital-based initiatives. For example, Vaughn et al^24^ found an increase in reported use of infection prevention practices against CDI in Veterans Affairs hospitals. However, these are survey-reported improvements, and compliance with simple practices, such as hand hygiene, is still reported as suboptimal worldwide.^25^

Second, our findings could reflect national improvements in antibiotic stewardship practices. Certain antibiotics, such as fluoroquinolones, second-generation and beyond cephalosporins, carbapenems, and clindamycin, are associated with an increased risk of immediate and prolonged CDI.^26,27,28^ Two meta-analyses^29,30^ found that antibiotic stewardship, by cycling and restricting exposure to high-risk antibiotics, was associated with a significant protective effect for HO-CDI. In 2017, the Infectious Diseases Society of America and Society for Healthcare Epidemiology of America clinical practice guidelines recommended the implementation of antibiotic stewardship practices and programs to battle CDI.^31^ In a cohort of US Southeastern hospitals, fluoroquinolone prescription declined between 2013 and 2017, and this reduction was even steeper after the FDA black box warning on fluoroquinolones (summer 2016),^32^ just before the implementation of VBIP for HO-CDI. In addition, the CDC reported a significant upward yearly trend in the percentage of national hospitals meeting all 7 core components of antibiotic stewardship since 2014.^14^ These antibiotic stewardship efforts also extend beyond the inpatient setting, and better outpatient antibiotic prescribing practices could partially account for the concurrent reduction in CO-CDI rates that we observed.^33,34^

A third potential explanation for the decline we observed in HO-CDI rates relates to implementation of better diagnostic stewardship by the health care facilities in our cohort.^35^ Solanky et al^36^ showed that diagnostic stewardship had the most substantial association with reductions in HO-CDI rates among other interventions, including prompt isolation of patients with CDI, optimal environmental cleaning, hand hygiene, fluoroquinolone and proton-pump inhibitor stewardship, and the rejection of solid stool samples by the laboratory. Several other studies that used diagnostic stewardship interventions significantly reduced unnecessary testing using a variety of approaches, such as health care professional education, decision-making algorithms, and the deployment of what are known as hard stops requiring a specialist consultation or laboratory approval prior to test ordering.^35,37,38,39,40,41^

Diagnostic stewardship may reduce the false-positive HO-CDI cases reported to NHSN due to laxative use, absence of significant diarrhea, and delayed testing that results in misclassification of CO-CDI as HO-CDI.^42,43^ Some studies have reported that CO-CDI rates are increasing or stable over the same period as our study^1,13,15^; however in our analysis, we found a commensurate decline in both VBIP-targeted HO-CDI and CO-CDI events. This suggests there was likely a decline in the total number of *C. difficile* tests conducted (both on admission and during hospitalization), which may be due to overall better diagnostic stewardship (ie, avoid patients on laxatives or those with no significant symptoms) rather than shifting HO-CDI to CO-CDI. Regardless of the mechanism behind HO-CDI temporal decline, this change is likely to be accompanied by lower costs and less overtreatment and exposure to unnecessary antibiotics because of false-positive *C. difficile* test results.^37^

### Strengths and Limitations

Our study has several strengths, including the use of prospectively collected data from the largest national database for CDI reporting. Additionally, the hospitals in our large sample are geographically and demographically representative of the United States. Our study also has several potential limitations. First, we did not have information on the number of CDI tests ordered for each hospital, which limited our ability to study changes in diagnostic stewardship. Therefore, we used other methods (ie, the ratio of reported HO-CDI to CO-CDI cases and the temporal trends in the percentage of NAAT testing) to indirectly draw conclusions on the association of diagnostic stewardship with the reduction in HO-CDI rates. Future CDI surveillance and evaluation could be enhanced by capturing data on the precise testing method used and the total number of tests performed. Second, hospital practices change over time, including the type of testing used and use of diagnostic or antimicrobial stewardship or other interventions. To address this issue, our GEE analyses were clustered at the hospital level, and we considered changes in the type of testing used over time in the model. Third, CO-CDI events (laboratory-identified events within the first 3 days of admission) used in our sensitivity analysis are captured by hospitals and may not be representative of CDI rates in the entire community. Fourth, our analysis was focused on facility-wide reported events, and we did not have data about patient characteristics that might reflect shifts in population risk of CDI over time. Fifth, the reported method of testing for each facility is classified only based on NHSN rule for the majority (>50%) testing method used; other methods that represent the principal minority could have been overlooked in our analysis. However, we did not observe significant shifts in the proportion of testing methods over time, suggesting that the impact of other testing methods was likely small. Sixth, we did not have a group of control hospitals that were not subject to VBIP, nor were we able to include patient-level data for risk adjustment. These factors limit our ability to draw conclusions on the causal association between VBIP implementation and the reduction in HO-CDI rates.

## Conclusions

The implementation of VBIPs was associated with a sustained decline in rates of HO-CDI, in contrast to previously reported VBIPs, which do not appear to have affected the rates of other targeted HAIs. The observed decline in HO-CDI rates may be due to greater opportunities for improvement in diagnostic and antimicrobial stewardship, which also align with the value these programs intend to achieve. Given that CMS payment policies have not previously been associated with improvements in other targeted HAI rates, future research should focus on elucidating the specific processes that contributed to improvement in HO-CDI rates to better inform the design of future interventions targeted by VBIPs.
